# Comparative three-dimensional connectome map of motor cortical projections in the mouse brain

**DOI:** 10.1038/srep20072

**Published:** 2016-02-02

**Authors:** Minju Jeong, Yongsoo Kim, Jeongjin Kim, Daniel D. Ferrante, Partha P. Mitra, Pavel Osten, Daesoo Kim

**Affiliations:** 1 Department of Biological Sciences, Korea Advanced Institute of Science & Technology, Daejeon, Korea, 305–338; 2 Cold Spring Harbor Laboratory, Cold Spring Harbor, New York, USA

## Abstract

The motor cortex orchestrates simple to complex motor behaviors through its output projections to target areas. The primary (MOp) and secondary (MOs) motor cortices are known to produce specific output projections that are targeted to both similar and different target areas. These projections are further divided into layer 5 and 6 neuronal outputs, thereby producing four cortical outputs that may target other areas in a combinatorial manner. However, the precise network structure that integrates these four projections remains poorly understood. Here, we constructed a whole-brain, three-dimensional (3D) map showing the tract pathways and targeting locations of these four motor cortical outputs in mice. Remarkably, these motor cortical projections showed unique and separate tract pathways despite targeting similar areas. Within target areas, various combinations of these four projections were defined based on specific 3D spatial patterns, reflecting anterior-posterior, dorsal-ventral, and core-capsular relationships. This 3D topographic map ultimately provides evidence for the relevance of comparative connectomics: motor cortical projections known to be convergent are actually segregated in many target areas with unique targeting patterns, a finding that has anatomical value for revealing functional subdomains that have not been classified by conventional methods.

The motor cortex plays an essential role in generation and control of movements^1^,^2^,^3^,^4^. Studies have revealed the anatomical basis of motor cortical functions: the motor cortex initiates muscular contractions through subcerebral projections to the brain stem and the spinal cord^5^,^6^,^7^. In addition, the motor cortex can mediate elaborate control of simple to complex behaviors through projections to the cortex and other subcortical areas, such as the striatum and the thalamus^8^,^9^,^10^.

The functional domain of the motor cortex is largely divided into the primary (MOp) and secondary (MOs) motor cortices which play central and modulatory roles, respectively^11^,^12^,^13^,^14^,^15^. In rodents, these subdomains are also known as the lateral (AGl) and medial (AGm) agranular cortices^16^,^17^, however the functional distinctions between them are not as clear^17^,^18^,^19^,^20^ as that in primates^11^,^14^. Although electrical stimulation of MOp and MOs can respectively evoke forelimb and whisker movements^18^,^20^, recent studies have provided evidences for potential roles of rodent MOs in cognitive motor control processes such as action sequence chunking^21^, action selection^22^, motor learning^23^ and planning^9^,^24^. These two subdomains send out projections to subcortical areas through two cortical layers—layer 5 (L5) and layer 6 (L6)^25^,^26^,^27^,^28^,^29^—thereby producing four motor cortical outputs. The circuit structure of these four motor cortical outputs may support the functional complexity of motor cortices.

Recent studies have extensively mapped the neural circuit structure in rodent brains^30^,^31^,^32^,^33^,^34^,^35^,^36^,^37^,^38^, showing an overall targeting pattern of motor cortical projections at a mesoscopic level. These studies suggest that MOp and MOs share substantial target areas in common^33^,^34^,^35^. In the neural circuit model, neural convergence is a critical network structure that leads to summation of more than two different signals to generate post-synaptic outputs, creating a computational module of neural information^39^,^40^,^41^,^42^. However, the neural convergences of the four motor cortical projections, which might yield critical information about the anatomical basis of motor signal processing and how the motor cortex modulates from simple to complex behaviors, have not been precisely compared. Here, we constructed a comparative three dimensional (3D) map of motor cortical projections using whole-brain serial two-photon (STP) tomography^43^ and cortical layer-specific Cre-transgenic mice^32^. This map provides insight into how four different motor cortical projections have actomic and topographic patterns in different and similar target areas.

## Results

### Mapping of layer-specific cortical projections derived from MOp and MOs motor cortical subdomains

**Figure 1:**
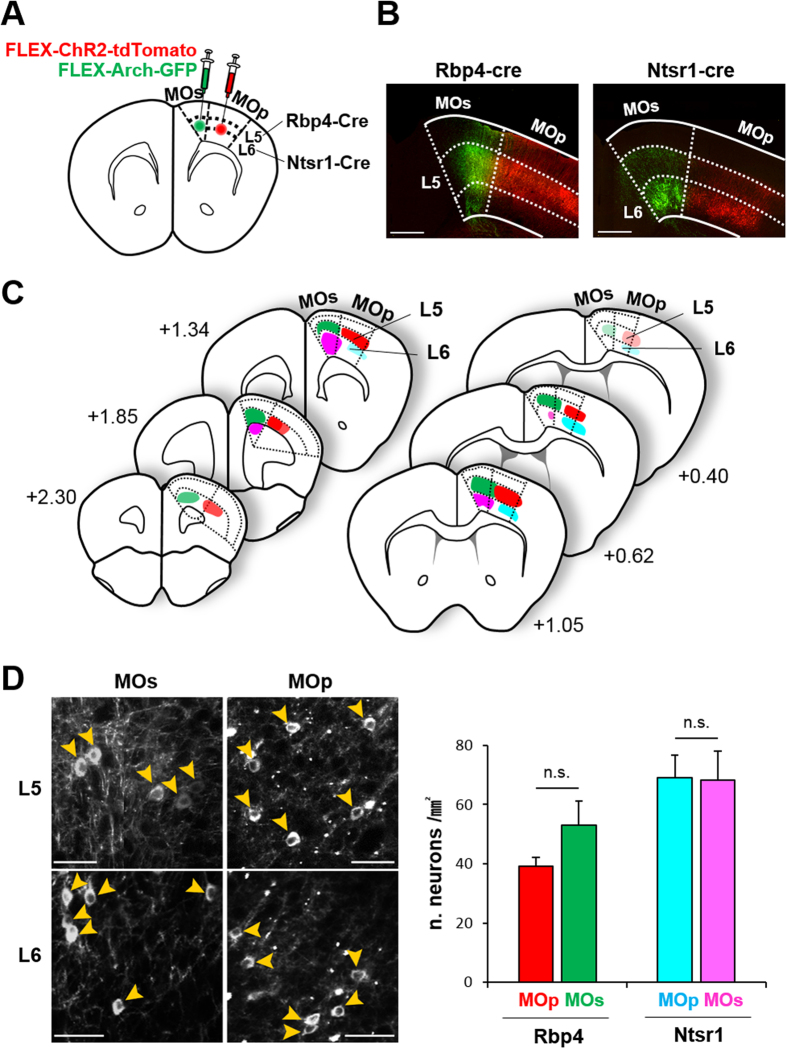
Targeting of layer- and subdomain-specific motor cortical neurons. (**A**) Scheme for viral tracer injection in Rbp4-Cre mice for layer 5 neurons and Ntsr1-Cre mice for layer 6 neurons in the MOp and MOs. (**B**) Representative fluorescent images of injection sites in the motor cortex of Rbp4-Cre (left) and Ntsr1-Cre (right) mice. Scale bar: 500 μm. (**C**) The extent of viral expressions in the motor cortex. Numbers represent the coordinates of the anterior-posterior axis from the bregma (mm). Red and Cyan represent tdTomato signals and Green and Magenta represent GFP signals. The opacity of each color indicates relative viral-expression levels, based on the data of averaged projection signals on RSTP brain (see also Supplementary Movie S1). (**D**) Counting of viral-infected neurons. Representative high-resolution images of virus infected motor cortical areas (left). Yellow arrows indicate the virus infected neurons. Scale bar: 50 μm. The number of virus infected cells per transfected areas (mm^2^) in each cortical subdomains of each transgenic line (right). There are no statistical significant differences between MOp and MOs in both Rbp4-Cre (L5) and Ntsr1-Cre (L6) lines. P = 0.147 for Rbp4-Cre (L5), n = 5; P = 0.938 for Ntsr1-Cre (L6), n = 5, paired t-test.

To visualize the projections of mouse MOp and MOs neurons, we injected two types of opsins conjugated with different chromophores, ChR2-tdTomato and Arch-GFP, into each subdomain (Fig. 1A). Using Rbp4-Cre and Ntsr1-Cre lines, which selectively express Cre recombinase in neuronal subpopulations confined to cortical layer 5 (L5) and layer 6 (L6)^32^,^44^, respectively, we successfully labeled output neurons from these two layers of MOp and MOs (Fig. 1B). Viruses were broadly transfected into the motor cortex, from 2.33–0.38 mm anterior to the bregma (Fig. 1C and Supplementary Table S1). To compare differences in expression levels between MOp and MOs of each transgenic line, we counted the number of virus infected cells per subdomain area and confirmed that there were no differences (Fig. 1D).

**Figure 2:**
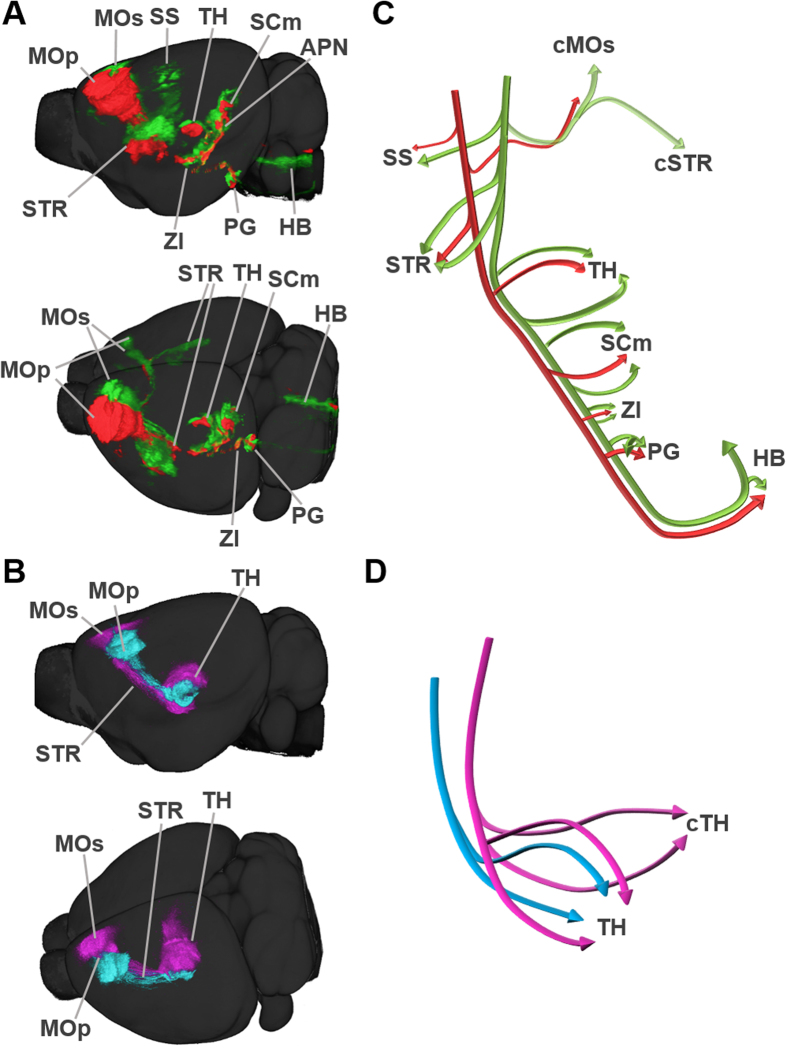
3D tractome maps of motor cortical projections. (**A,B**) Representative Rbp4-Cre for labeling L5 (**A**) and Ntsr1-Cre for L6 (**B**) brains registered to the RSTP. (**C,D**) Schematic depiction of neural tracts and projections from L5 (Rbp4-Cre) (**C**) and L6 (Ntsr1-Cre) (**D**) neurons in the motor cortex. Projections from L5 cortical neurons to other areas were omitted for simplicity. GFP and tdTomato signals in Ntsr1-Cre mice are pseudo-colored as magenta and cyan, respectively. STR, striatum; cSTR, contralateral striatum; cTH, contralateral thalamus; HB, hindbrain. Other abbreviations are presented in Table 1.

Whole-brain STP tomography^43^ was used to obtain a well-aligned series of 2D images with 1 × 1 μm^2^ *xy* resolution in every 50 μm *z* sampling (Supplementary Fig. S1A; all 2D images were presented on the website http://mouse.brainarchitecture.org/celltypes/connectivity/) that could be easily assembled into a 3D representation. Each brain sample was registered to a reference STP (RSTP) aligned with the Allen Reference Atlas for anatomical segmentation (Supplementary Fig. S1B)^45^. The projection signals were normalized to the number of virally labeled cells in the injection area, and mapped onto the RSTP using an image registration parameter (Fig. 2A,B). This procedure allowed projection pattern data obtained from different brains to be quantitatively compared in the same standard space (n = 5 brains per Cre line for each brain area).

An analysis of the resulting 3D projection images revealed that L6 neurons in Ntsr1-Cre mice send projections exclusively to the thalamus, whereas L5 neurons from Rbp4-Cre mice send to other cortical and subcortical areas, including the striatum, hypothalamus, midbrain and hindbrain, as well as the thalamus (Fig. 2, Table 1, and Supplementary Movie S1), as previously reported^25^,^26^,^27^,^32^. Tracts from MOp and MOs neurons of both cortical layers maintain their relative positions, whereas their projections are diversely distributed (Fig. 2 and Supplementary Movies S2,S3). These projection patterns were highly reproducible within five different samples obtained for each layer and cortical region (Supplementary Fig. S1C).

### Quantitative comparison of layer-specific cortical projections between motor cortex subdomains

Relative projection strength may also provide clues about corresponding neural network properties, yielding insight into their functional roles as well as their topographical organization^34^,^46^. A quantitative comparison of MOp and MOs projections on the axon topography map (Table 1 and Supplementary Movie S1) revealed a number of differences. First, the MOs in general sends projections to more diverse regions, including the ipsilateral and contralateral side of the cortex, striatum, thalamus, and hindbrain. For example, axons from L5 MOs neurons are found in many higher cognitive cortical regions, such as the lateral (ORBl) and ventral lateral (ORBvl) orbital cortex, dorsal (AId) and ventral (AIv) agranular insular cortex, posterior parietal association area (PTLp), temporal association area (TEa) and ectorhinal area (ECT), whereas MOp neurons project to none of these areas (Table 1 and Supplementary Movie S1). L5 MOs neurons also project bilaterally to amygdala nuclei, specifically the anterior basolateral amygdala (BLAa) and capsular central amygdala (CEAc) (Table 1 and Supplementary Movie S1). Second, the MOs sends denser projections than the MOp. This feature is especially conspicuous in MOs outputs from L6 compared with those from L5 in the thalamus (Table 1 and Supplementary Movie S1). Third, despite the fact that both MOp and MOs L5-derived neurons project to a similar number of subcortical target areas in the hypothalamus and midbrain, the projection degree of L5 MOp projection tends to be less than that of L5 MOs in overlapping target areas (Table 1). Collectively, this quantitative anatomical evidence indicates that the MOs could be involved in more complex motor behaviors, such as motor planning^9^,^24^, compared with the MOp. Fourth, target areas generally receive a higher degree of cortical projections on the ipsilateral than the contralateral side. An exception to this is the AId and claustrum (CLA), which are more heavily appeared in the contralateral side (Table 1 and Supplementary Movie S1), as observed in rats^31^. The contralateral side of the thalamus also receive less MOp projections, similar in the previous reports^31^ however, they are ruled out in this quantitative data.

### Topographic organization in the thalamus

**Figure 3:**
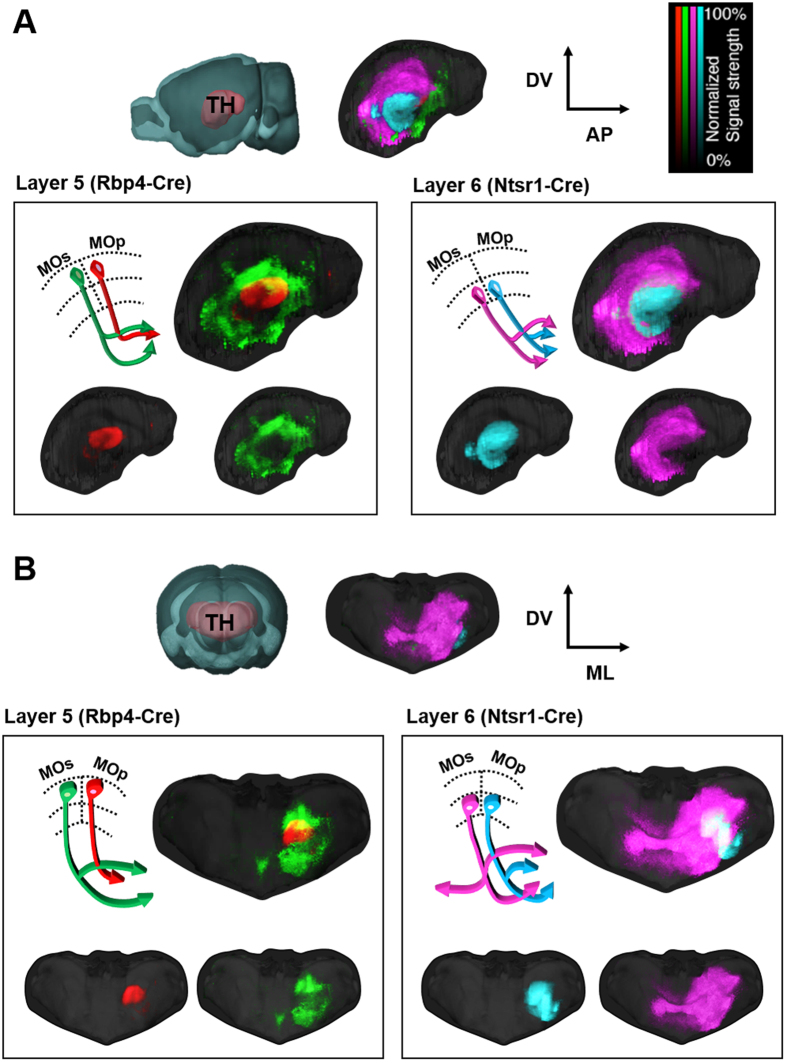
Comparative 3D topography patterns of motor cortical outputs from four subdomains to the thalamus. 3D sagittal (**A**) and coronal (**B**) views of the four different, averaged projection patterns in the thalamus. Red area in the reference brains indicates examined thalamic areas (TH). A, anterior; P, posterior; M, medial; L, lateral; D, dorsal; V, ventral. The heatmap represents normalized signal strength per voxel.

Next, we compared the projection patterns of cortical neurons within the same targets, especially the thalamus. Interestingly, MOp and MOs outputs from L5 and L6 were segregated, exhibiting different patterns of spatial organization (Supplementary Movies S1–S3). First, MOp projections form an interior core in the thalamus, whereas MOs projections form a shell around them (Fig. 3A). This core-capsular relationship between MOp and MOs projections is found in both L5 and L6 corticothalamic (CT) neurons, but with different patterns: for neurons in L5, MOs projections completely envelop those from the MOp (Fig. 3A, left), whereas L6 MOs projections enclose just the anterior portion of L6 MOp projections (Fig. 3A, right). Second, L5 outputs from both MOp and MOs primarily target the medial part of the thalamus, whereas L6 outputs are diffusely and broadly distributed over lateral regions (Fig. 3B).

**Figure 4:**
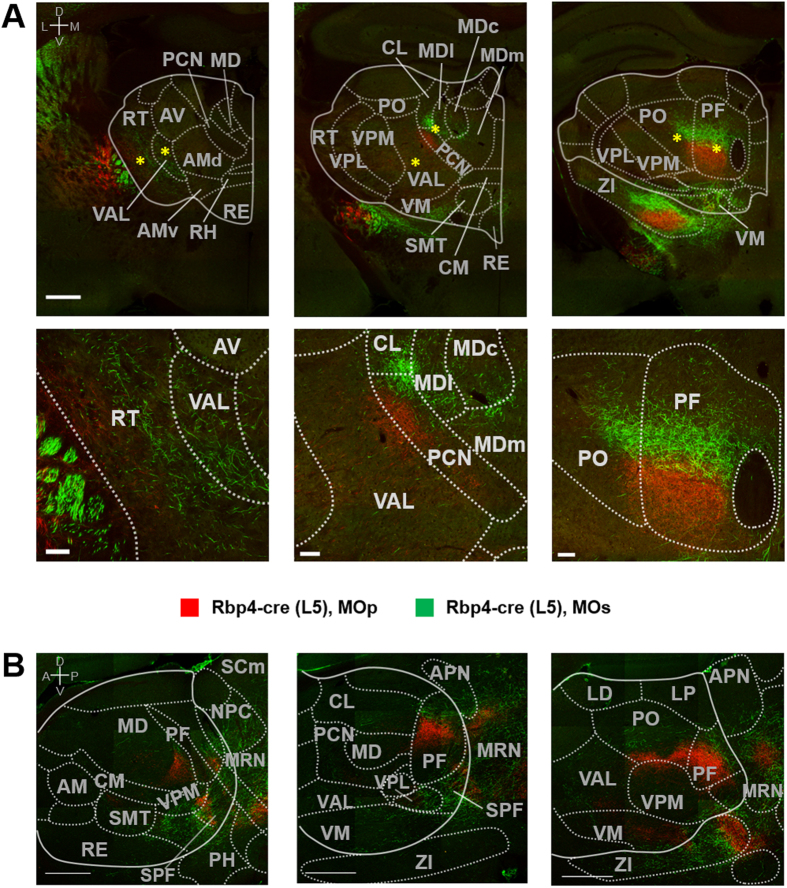
2D topographical organizations between MOp and MOs projections from L5 of the Rbp4-Cre mouse in the thalamus. (**A**) Representative coronal view images. Bottom panels are zoomed, high-resolution images of areas in top panels marked by asterisks. Coordinates are 0.76 mm, 1.30 mm, 2.26 mm posterior to bregma from left to right panels. Scale bars: 1 mm (top) and 100 μm (bottom). (**B**) Representative sagittal view images. From left to right, 0.48 mm, 0.72 mm, 0.84 mm lateral to bregma. Scale bars: 500 μm. Abbreviations are presented in Table 1. A, anterior; P, posterior; M, medial; L, lateral; D, dorsal; V, ventral.

**Figure 5:**
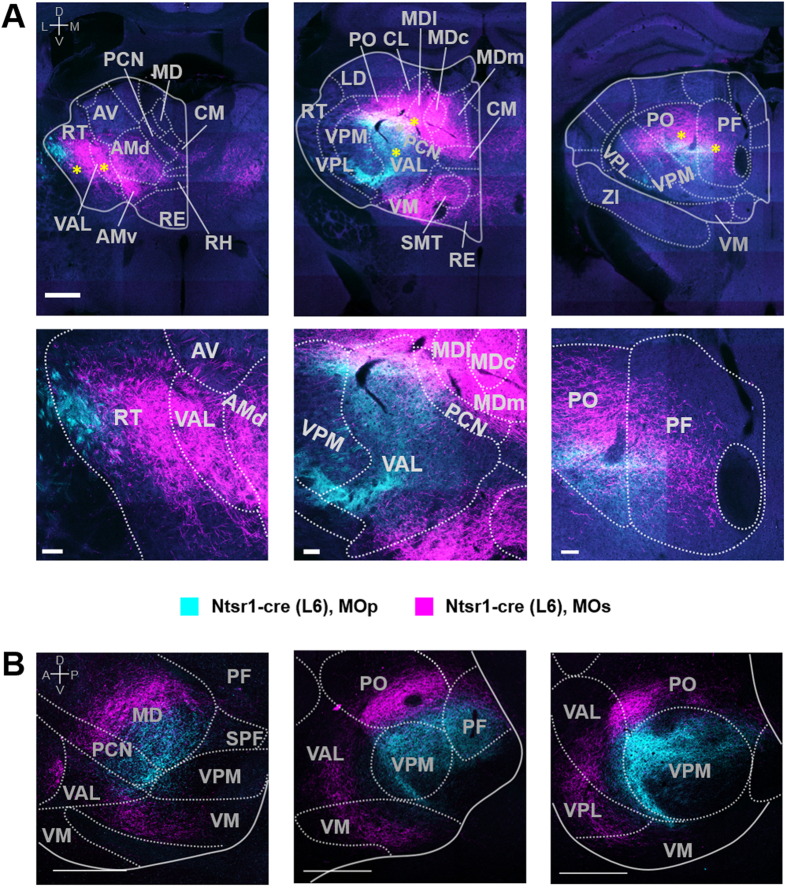
2D topographical organization of MOp and MOs projections from L6 of the Ntsr1-Cre mouse in the thalamus. (**A**) Representative coronal view images. Bottom panels are zoomed, high-resolution images of areas of top panels marked by an asterisk. Coordinates are 0.76 mm, 1.30 mm, 2.26 mm posterior to bregma from left to right panels. Scale bars: 1 mm (top) and 100 μm (bottom). (**B**) Representative sagittal view images. From left to right, 0.60 mm, 0.84 mm, 1.08 mm posterior to bregma. Scale bars: 500 μm. Magenta and cyan are pseudo-color representations of GFP and tdTomato signals, respectively. Abbreviations are presented in Table 1. A, anterior; P, posterior; M, medial; L, lateral; D, dorsal; V, ventral.

**Figure 6:**
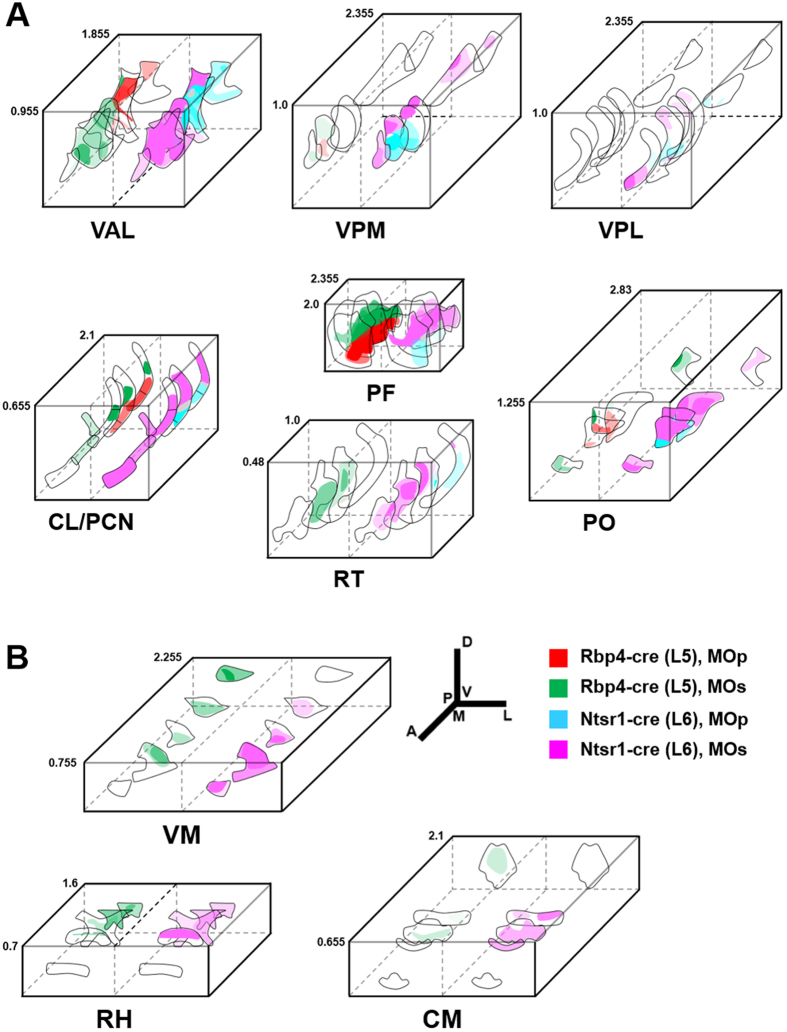
Projection-based parcellation map in the thalamus. Each thalamic nucleus and MOp and MOs corticothalamic inputs from L5 (Rbp4-Cre, left) and L6 (Ntsr1-Cre, right) are depicted as 3D illustrations. The opacity of each color represents the relative projection strength, which was based on the data of normalized and averaged projection signals on RSTP brain (see also Supplementary Movie S1). Please note the topographical segregation and varied projection strengths within the anatomical regions. Numbers on the side represent coordinates of the anterior-posterior position from the bregma. A, anterior; P, posterior; M, medial; L, lateral; D, dorsal; V, ventral. Abbreviations are presented in Table 1.

The thalamus consists of several subnuclei, and each target thalamic nucleus receives cortical projections with distinct features. Parcellation by domain- and layer-specific cortical projections are obviously observed in ventral and intralaminar thalamic nuclei groups and the posterior complex thalamus (PO) (Figs 4, 5, 6 and Supplementary Movie S1), known to be involved in motor function through interactions with the frontal cortices and the basal ganglia^47^,^48^,^49^,^50^,^51^,^52^. The parcellation patterns of MOp and MOs projections reveal different spatial relationships in each thalamic subnuclei (Figs 4, 5, 6). For example, in the ventral anterior-lateral (VAL) and paracentral (PCN) thalamic nuclei, axons from both L5 and L6 MOs neurons are located on the anterior, whereas axons from MOp appear at the posterior portion (Figs 4, 5, 6A and Supplementary Movie S1). Within PCN nuclei, where both MOs and MOp axons are observed, parcellation patterns are also appeared along a dorsal-ventral (DV) axis—MOs neurons project to a more dorsal part of the PCN compared to MOp (Figs 4, 5, 6A). In the central lateral (CL), posterior (PO) and parafascicular (PF) thalamic nuclei, MOs and MOp projections segregate along the DV axis (Figs 4, 5, 6A). This parcellation pattern clearly appears at the posterior part of the CL and the anterior part of the PF, while the rest of the CL and PF mainly receive MOs projections. MOs and MOp axons from only L6 exhibit the DV spatial relationship in the ventral posterior complex of the thalamus (VPM/VPL) (Figs 5 and 6A) and anterior-posterior/medial-lateral relationship in the reticular thalamic nucleus (RT) (Figs 5 and 6A).

The topographical organization patterns can also be observed between cortical layers. L6 neurons in the MOs constitute a more anterior part of the ventral medial (VM), central medial (CM) and rhomboid (RH) thalamic nuclei than L5 neurons in the MOs (Fig. 6B and Supplementary Movie S1). Although the mediodorsal thalamic nuclei (MD) are not clearly parcellated by L5 and L6 projections, L6 MOs projections do extend to the medial part of the MD (MDm) compared to L5 projections (Figs 4 and 5).

**Figure 7:**
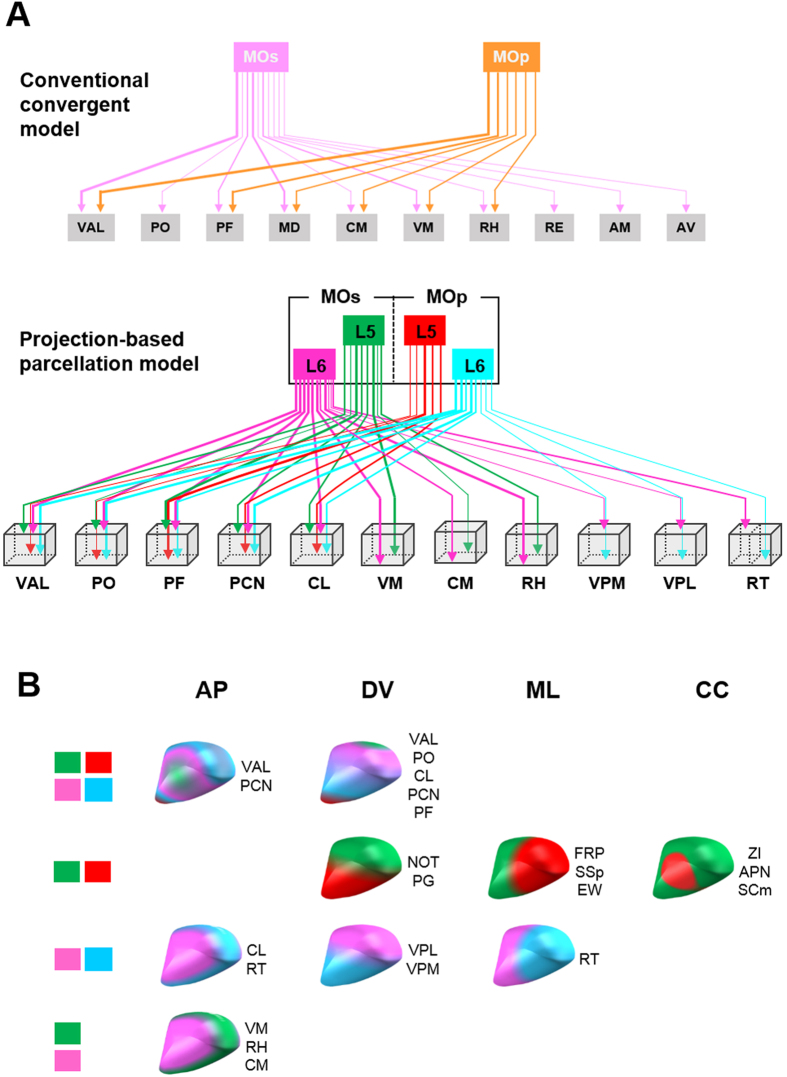
Summary of topography patterns. (**A**) The upper panel represents a conventional wiring diagram of connections from motor cortical areas to thalamic nuclei based on a previous study, summarized as a simple algorithmic convergent model^34^. The bottom panel is a proposed connectivity diagram considering projection-based parcellation patterns based on the present results. The tips of arrows indicate the relative spatial positions. The thickness of arrows in both panels indicates the degree of projection; quantitative data are shown in Oh *et al*. (2014) and Table 1. Connections from the motor cortex to other brain regions are omitted for simplicity. (**B**) 3D illustrations summarizing topographic segregation patterns of cortical outputs to target nuclei, showing spatial relationships along the anterior-posterior (AP), dorsal-ventral (DV) and medial-lateral (ML) axis, and the core-capsular (CC) pattern. Thalamic nuclei belong to each category were indicated next to the 3D schema. Color-coding is the same as the bottom panels of (**A**). Abbreviations of all brain regions are presented in Table 1.

Based on the serial two-photon imaging data, we could construct a 3D map of domain- and layer-specific cortical projections in each thalamic nucleus represented as schematic illustrations (Fig. 6). We also summarized this information in a 3D connectivity diagram from the cortex to the thalamus in Fig. 7A. Taken together, these results suggest that complex motor information can be processed through cortical projection-based modules of thalamic nuclei (Fig. 7A), each of which also consists of divergent or convergent neural networks with other sensory and associative cortical areas^53^.

### Topographic organization in other subcortical areas

Topographical organizations of MOp and MOs outputs were also found in other cortical and subcortical areas. For example, L5 MOp and MOs projections are segregated along the DV axis in the subthalamic nucleus (STN), nucleus of the optic tract (NOT) and PG (Supplementary Fig. S2A–C and Supplementary Movie S1). L5 MOp axons are found more dorsally than L5 MOs in the STN in contrast, the relationships are vice versa in the NOT and PG. In the frontal pole (FRP), primary somatosensory cortex (SSp) and Edinger-Westphal nucleus (EW), L5 MOp projections are appeared more laterally compared to L5 MOs (Supplementary Movie S1). The core-capsular spatial relationship observed in a view of the whole thalamus (Fig. 3) was also found in other subcortical areas: L5 MOs projections are found around core areas inside L5 MOp projections within the zona incerta (ZI), the anterior prectal nucleus (APN) and motor-related superior colliculus (SCm) (Supplementary Fig. S2D–F, and Supplementary Movie S1). However, segregation patterns of L5 MOp and MOs projections were not clear in other subcortical target areas (Supplementary Movie S1). This suggests that even the cortex, midbrain and brainstem, as well as the thalamus, can consist of complex structural modules formed by the projection-based parcellation of L5 motor cortical neurons.

## Discussion

Remarkably, the four motor cortical projections show spatially segregated patterns of innervation into even commonly convergent target areas of the whole brain (Figs 4, 5, 6, Supplementary Fig. S2 and Supplementary Movie S1). Moreover, these segregation patterns vary depending on brain region (Fig. 7B). Our findings suggest that brain connectivity is more sophisticated, and is systematically organized by segmented modules. Therefore, connectome modalities must be considered in this complex projection-based parcellation rather than just simple algorithmic convergent model (Fig. 7A). Doing so could help understand the function of whole brain neural networks.

Both L5 MOp and MOs projections have many common targets including the basal ganglia, midbrain, and medulla (Table 1 and Supplementary Movie S1). Despite this convergent targeting, L5 MOp and L5 MOs projections show different patterns of spatial organization within the same target nuclei (Figs 2A,C, 3 and 4, and Supplementary Fig. S2). From this we infer that these two projections could generate different combinations of muscle movements^54^ directly or indirectly activating different ensembles of motor neurons, although additional experimental evidence is required to confirm this.

In contrast, MOp and MOs neurons from both cortical layers exhibit more divergent connectivity patterns in the other brain regions such as the cortical areas and the thalamus (Table1 and Supplementary Movie S1). Notably, MOs projections target medial/intralaminar thalamic nuclei (Fig. 3 and Table 1), which are known to interact with prefrontal areas associated with working memory, perception, and sensory-guided movements^55^,^56^,^57^,^58^,^59^. Among MOs neurons targeting the medial/intralaminar thalamus, those derived from L6 exhibit denser and broader projection patterns than those in L5 (Figs 3, 4, 5 and Table 1). This suggests that cognitive motor functional roles of MOs^9^,^21^,^22^,^23^,^24^ may be mainly mediated through L6 MOs projections that communicate with frontal areas.

In the thalamus, L5 CT axons show spatially concentrated projections, whereas L6 CT axons show spatially dispersed projection patterns (Fig. 3B and Supplementary Fig. S3). Such anatomical aspects may be consistent with the properties of L5 and L6 as drivers transmitting information and modulators altering transmission of driver inputs, respectively^60^,^61^,^62^. Diffuse projection patterns of L6 CT neurons seem to be more effective for the gradual way of modulation, rather than all-or-none relays of neural signals, although these correlations remain to be investigated. The precise topographic map between L5 and L6 projections into the thalamus (Fig. 6 and Supplementary Movie S1) in this study provides basic anatomical information that should effectively aid in further investigation of the functions of CT neurons as drivers and modulators in motor behavior.

Although the sensory functions of the thalamus in physiological^63^,^64^ and pathological^65^ conditions have been well documented, motor functions of the thalamus and their regulation by CT projections are not fully understood^66^, largely owing to the complexity of relevant anatomical connections. Our CT projection map (Figs 4, 5, 6 and Supplementary Movie S1), taken together with a recently reported thalamocortical (TC) projection map^67^, can elucidate interaction patterns of thalamic nuclei with the cortex further considering cortical layers. For example, VAL, PO and PCN receive CT projections from both L5 and L6 in the motor cortex (Figs 4, 5, 6 and Table 1); VAL and PCN thalamic neurons project to L5b, whereas PO thalamic neurons project to L2/3-L5a of the motor cortex^67^. PF thalamic neurons receive dense cortical inputs but send fewer outputs to the motor cortex; this projection pattern is opposite in the lateral posterior thalamic nucleus (Table 1)^67^.

In the VAL, dense projections from MOp CT neurons exit laterally, whereas most TC neurons projecting to the MOp are located medially^68^. These anatomical findings are consistent with electrophysiological results showing weak-to-absent direct monosynaptic connections between the MOp and VAL^68^. In addition, TC neurons in the medial part of VAL projecting to the MOp^68^ appear to receive cortical projections derived from the MOs (Fig. 5A and Supplementary Movie S1). Based on the collective findings, we hypothesize that MOs may modulate MOp neurons through the motor thalamus. Furthermore, the detailed topographic maps of cortico-thalamo-cortical loops^67^ (Figs 4, 5, 6 and Supplementary Movie S1) should aid in understanding their function in sensorimotor regulation.

This 3D tractome map can be used to address a number of issues in addition to that described above. First, spatial parcellation into hub areas by MOp and MOs outputs as shown in our research (Fig. 7) can be used as additional criteria to distinguish between the two subdomains of the rodent motor cortex. Second, the boundaries between thalamic nuclei as determined by cytoarchitectonics are likewise not clear^67^,^69^. In this context, a previous study suggested that patterns of thalamocortical input could be used to identify thalamic nuclei^67^; specific combinations of cortical targets could be used as a standard for defining thalamic subdivisions. The projection-based parcellation patterns presented here indicate that each thalamic nucleus can be readily divided according to the unique distribution patterns of cortical projections (Fig. 6), potentially providing more precise information regarding the identity of specific thalamic nuclei.

The present study compared the topographic projection patterns of motor cortices especially to subcortical targets, via layer-specific expression of chromophores. However, our findings did not distinguish neuronal subtypes of L5^32^, namely the pyramidal tract (PT)-type projecting to the spinal cord with collaterals to other brain regions and IT-type neurons projecting to only the cortex and striatum. In addition, our study was restricted to CT neurons in L6, which is also known to contain IT-type neurons^70^, due to specific labeling by Ntsr1-Cre^32^. These limitations may lead to underestimation of the degree of cortical projections from L5 neurons, compared to L6. We believe that more experiments with other layer- and cell type-specific Cre-driver mice^32^ may help to solve the above issue and elucidate the full topographic map of mouse brain. Further studies are additionally needed to address whether one cortical neuron sends axons to a single subcortical target nucleus or simultaneously to several target nuclei, as shown previously in rats^71^.

### Concluding remarks

Knowledge of connectivity patterns among brain regions makes it possible to infer which types of interactions are possible^72^. The 3D tractome map presented here describes inter-circuit relationships especially in convergent target areas, which may prove critical for the prediction of function. A similar approach to establishing topographical relationships for other cortical projections will be useful for investigations into structural bases of neural circuit function.

## Materials & Methods

### Animals

All animal care and experimental procedures were performed in accordance with protocols approved by the directives of the Animal Care and Use Committee of Korea Advanced Institute of Science and Technology (approval number KA2012-04). Rbp4-Cre (No. 031125-UCD, MMRRC) and Ntsr1-Cre (No. 017266-UCD, MMRRC) mice were generated by mating the respective heterozygous animals (C57BL/6J backgrounds). Mice were maintained under a 12:12 hour light:dark cycle with free access to food and water.

### Stereotaxic viral injections

Male mice (8–16 weeks old) were anesthetized with 2,2,2-tribromoethanol (20 mg/ml, i. p.; Sigma T48402) and placed on a stereotaxic apparatus (Kopf Instruments) for surgery. All injections were performed with a 10-μl syringe (Hamilton Company), guide cannulas (33-gauge NanoFil Needle Assortment, blunt; World Precision Instruments), and a syringe pump (KD Scientific). The MOp injection coordinate was 1.55 mm anterior, −1.95 mm lateral, 0.8 mm (L5) or 0.9 mm (L6) ventral to bregma. The MOs injection coordinate was 1.55 mm anterior, −1.0 mm lateral, 0.8 mm (L5) or 0.9 mm (L6) ventral to bregma. We targeted a particular point at the center of each cortical subdomains based on the Allen reference atlas (http://atlas.brain-map.org) to broadly transfect viruses from anterior to posterior motor areas (Fig. 1C). The coordinate information about mean ranges of virus-expressing cortical areas are in the Supplementary Table S1. 0.6 μl of recombinant AAV2/1.CAGGS.FLEX.ChR2.tdTomato.SV40 (5.0 × 10^12^ particles/ml; University of Pennsylvania) or AAV2/2.CAGGS.Flex.Arch.GFP (5.0 × 10^12^ particles/ml; University of North Carolina Vector Core) were injected into the MOp and MOs, respectively, at a rate of 0.065 μl/min. For sagittal view images (Figs 4B and 5B), 0.6 μl of recombinant AAV2/9.EF1α.dflox.hChR2(H134R).mCherry.WPRE.hGH (2.0 × 10^12^ particles/ml; University of Pennsylvania) and AAV2/2.EF1α.DIO.eArch3.0.eYFP (2.0 × 10^12^ particles/ml; University of Pennsylvania) were injected into the MOp and MOs, respectively. The guide cannula was removed 5 minutes after completion of the injection.

### Tissue preparation and STP tomography imaging

Two weeks after viral injection, animals were deeply anesthetized with 2,2,2,-tribromoethanol, killed, and then fixed by transcardiac perfusion with 4% paraformaldehyde (PFA; diluted in 0.2 M phosphate buffer) after being perfused with 0.9% saline. Brains were removed and post-fixed in 4% PFA overnight (<24 hours) at 4 °C. The solution was changed to 0.1 M glycine in 0.1 M phosphate buffer, and the incubation period was extended for two more nights at 4 °C. Fixed brains were stored in 0.1 M phosphate buffer at 4 °C until imaged. The serial two-photon (TissueCyte 1000; Tissuevision) imaging procedure used has been previously described in detail^43^. Briefly, the brain was embedded in 4% oxidized agarose and cross-linked with a 0.2% sodium borohydrate solution (in 0.05 M sodium borate buffer, pH 9.0–9.5). The entire brain from the olfactory bulb to the end of the cerebellum was imaged as 12 × 16 × 280 (*xyz*) tiles with a 1 × 1 μm^2^ *xy* resolution for every 50 μm *z*. A wavelength of 970 nm was chosen for two-photon excitation to efficiently excite both red and green signals. To efficiently separate green from red signals, we used 560 nm dichroic mirror (Chroma, T560LPXR) and band path filters (Semrock FF01-607/70-25 for red and Semrock FF01-520/35-25 for green signal). The resulting tiles in each channel were stitched with custom-built software, and 3D rendering was done using the Volocity software (Perkin Elmer). There was no signal bleedthrough (contamination from another signal) except virus-infected cell bodies.

### Sectioning and confocal imaging

For conventional histological examination, 40-μm–thick sagittal brain sections were cut on a vibratome (VT1200S, Leica) after perfusion and post-fixation in 4% PFA overnight. Slices were mounted onto glass slides with Vectashield mounting medium containing 4′,6-diamidino-2-phenylindole (DAPI, H-1200; Vector Labs). Sagittal views of the thalamic nucleus were imaged as 3 × 3 (*xy*) tiles with an LSM780 confocal microscope (Carl Zeiss) equipped with a Plan-Apochromat 10x/0.45 M27 objective.

### Projection data processing

Previously published methods were adopted for quantifying neuronal projections as imaged by STP tomography^34^. In brief, filter images of the original image data were generated by applying a square root transformation, histogram matching to the original image, and median and Gaussian filtering using ImageJ (NIH) software. The original images were then subtracted from the filtered images to generate signal images. These were then converted to binary maps by applying a threshold chosen to maximize signal retention while minimizing background autofluorescence. We cannot rule out the possibility that very faint and sparse signals were being missed in our automatic detection. The number of binary signals in each evenly spaced, non-overlapping 20 × 20 μm^2^ (xy dimension) area was calculated and assigned as a value for the corresponding voxel across the entire brain; this value represented the “projection strength” (percentage of signal pixels) in the given space. It is important to note that the current signal detection method cannot distinguish axon terminals from passing fibers. We also manually removed false-positive signals from bleed-through in the injection site and hyper-intense, non-specific signals from the dura. Mutual information from background autofluorescence was used to compute image registration parameters to fit each brain to an RSTP brain using Elastix^73^, based on a previously described method^45^. This was then used to transform the projection signal data in each voxel to the RSTP brain. For normalization of projection signals, first the projection strength from each brain was divided by manually counted virus-infected cells in injection areas from the corresponding brain (Fig. 1D). Second, the normalized signal was multiplied by the average value of cell counts from 20 injections (2 injection sites × 2 layer-specific Cre lines × 5 samples) to make the projection strength in original quantification scale. The normalized projection signals from each kind were then averaged to generate a projection voxel map for each injection site (MOp or MOs) for each Cre mouse line (Ntsr1-Cre and Rbp4-Cre) (Supplementary Movie S1). To calculate how much area was occupied by projection signals in each region of interest (ROI), we counted the number of voxels containing signals in each anatomical RSTP labels, divided it by the total number of voxels in each ROI, and multiplied by 100 to represent the number as percentage. We termed this measurement as a “projection degree” (Table 1) that represent how much given anatomical areas are occupied by projection signals. Negligible projection with less than 1% in the projection degree was considered “None” for simplicity. Pearson correlation test was used to assess projection reliability between different injections based on projection degrees in each anatomical ROI across all samples (Supplementary Fig. S1C).

## Additional Information

**How to cite this article**: Jeong, M. *et al*. Comparative three-dimensional connectome map of motor cortical projections in the mouse brain. *Sci. Rep.* **6**, 20072; doi: 10.1038/srep20072 (2016).

## Supplementary Material

Supplementary Movie S1

Supplementary Movie S2

Supplementary Movie S3

Supplementary Information

## Figures and Tables

**Table 1:**
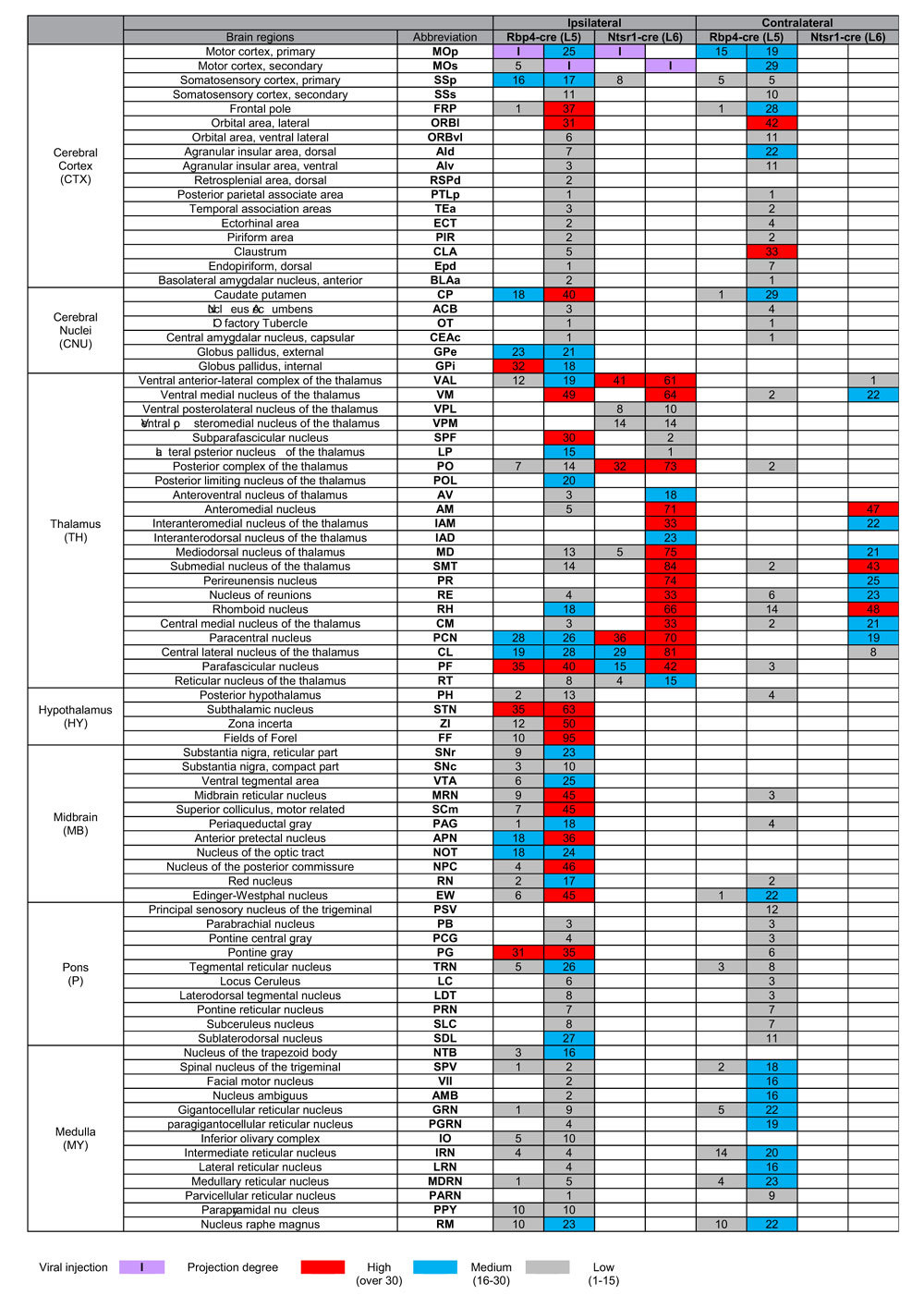
Whole-brain projection patterns and degrees in Rbp4-Cre (L5) and Ntsr1-Cre (L6) mice.

Averaged projection signals (n = 5 brains per kind) after being normalized by infected cells were used to determine how much of the area of each anatomical region of interest (ROI) is occupied by the signal, termed as projection degree. Projection degree is the percentage of areas occupied by the projection signal divided by total area (see Materials and methods for more details). Grey, blue, and red color are used for areas with projection degree with 1–14.9, 15–29.9, and 30 and more, respectively. Less than 1% in the projection degree was considered “None” for simplicity. Anatomical ROIs are ordered based on ontology presented in the Allen Brain Atlas (http://atlas.brain-map.org/).
