# Supplementary material for: Comparative three-dimensional connectome map of motor cortical projections in the mouse brain
Source: Sci Rep. 2016 Feb 2;6:20072. doi: 10.1038/srep20072 (PMC4735720; doi:10.1038/srep20072)
Supplement: Supplementary Information [file srep20072-s4.pdf]

Comparative three-dimensional connectome map of motor cortical projections in the mouse brain

Minju Jeong[1*], Yongsoo Kim[2,3*], Jeongjin Kim[1], Daniel D. Ferrante[2], Partha P. Mitra[2], Pavel Osten[2], Daesoo Kim[1†]

1 Department of Biological Sciences, Korea Advanced Institute of Science & Technology, Daejeon, Korea, 305-338

2 Cold Spring Harbor Laboratory, Cold Spring Harbor, New York, USA

3 Present address: Department of Neural and Behavioral Sciences, College of Medicine, Penn State University, Hershey, PA, USA

* These authors contributed equally to this work.

† Correspondence addressed to

Daesoo Kim, PhD

Department of Biological Sciences

Korea Advanced Institute of Science and Technology (KAIST)

291 Daehak-ro, Yuseong-gu

Daejeon 305-338, Republic of Korea

Phone: 82-42-350-2639

Fax: 82-2-350-5639

Email: daesoo@kaist.ac.kr

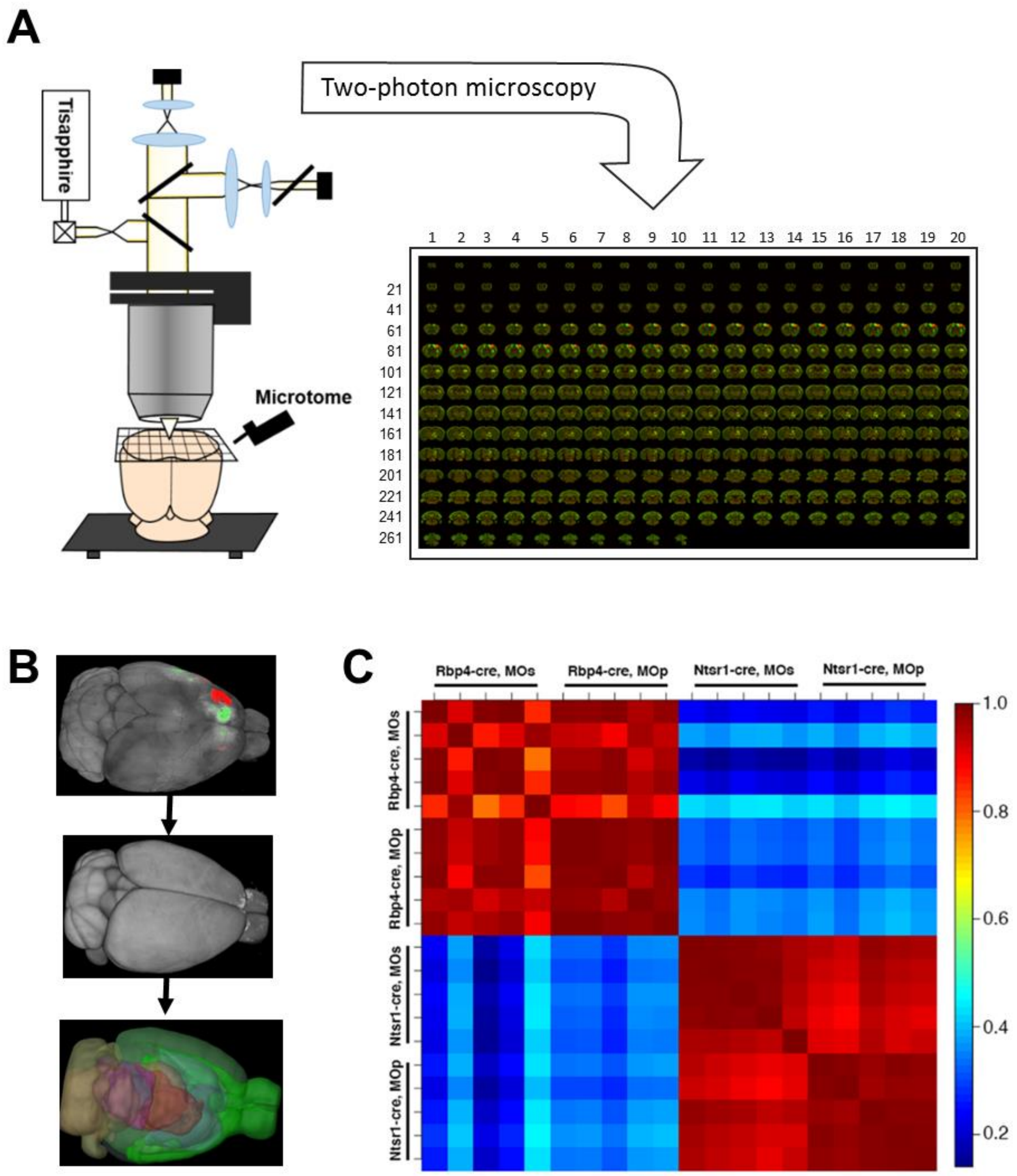

Supplementary Figure S1. Mapping of layer- and subdomain-specific motor cortical neurons. (A) Sample 270-serial sections (50-μm z-spacing between sections) of the virus-injected brain (right) imaged by STP tomography (left). All images were displayed on the website (http://mouse.brainarchitecture.org/celltypes/connectivity/

). (B) STP 270-serial z-sections were reconstructed to yield a 3D image (upper). Each 3D brain was registered to the RSTP (middle) aligned with the Allen Reference Atlas (bottom). (C) Highly reproducible cortical projection patterns within different samples. Pearson correlation of projection patterns between Rbp4-Cre and Ntsr1-Cre brains (n = 5 samples each), showing MOs and MOp signals from each Cre-line, presented as heatmaps of R-values; average R-value for brains in the same group = 0.96 ($p < 0.01$, one-tailed test).

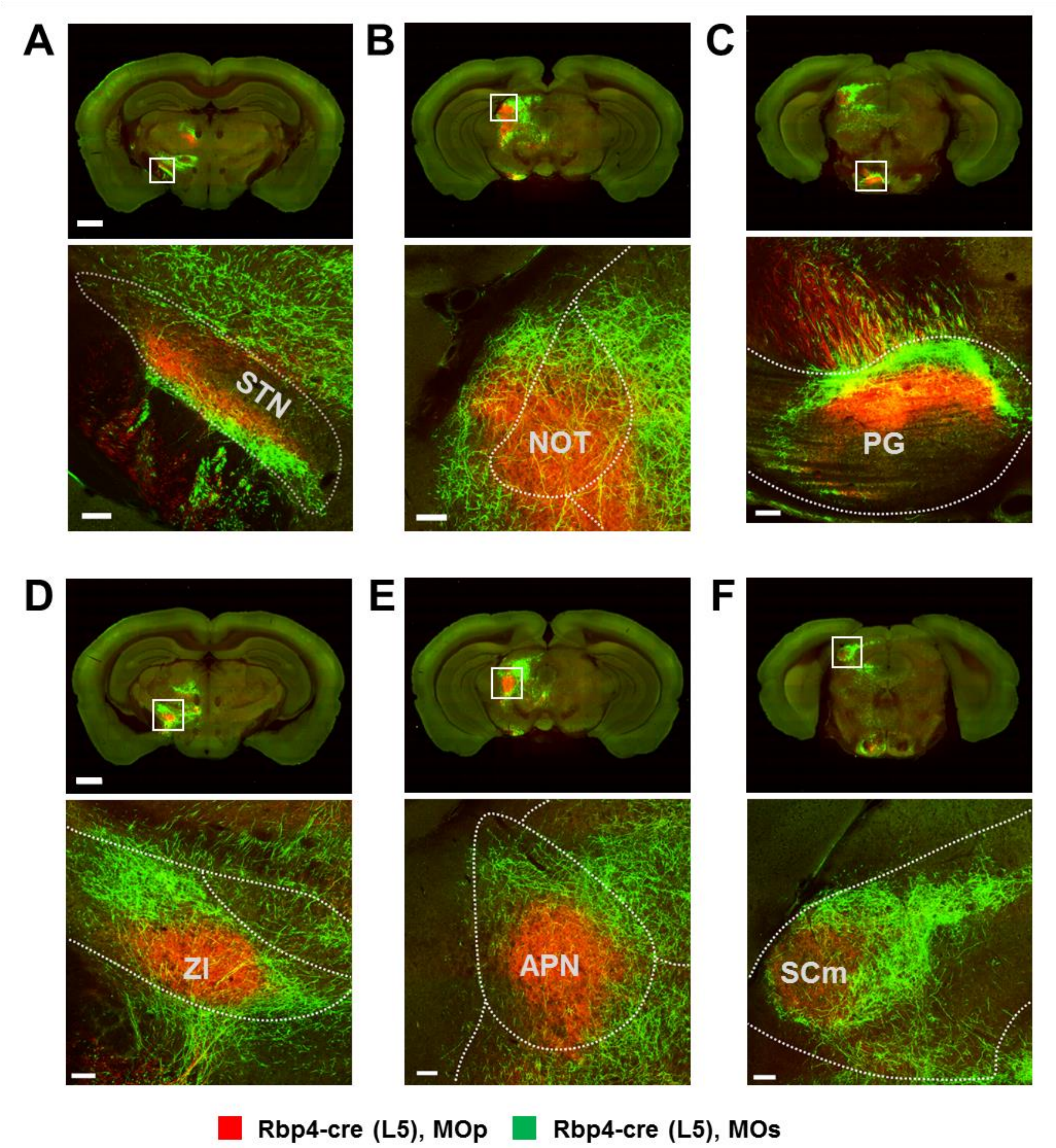

Supplementary Figure S2. Segregated topographic patterns of L5 MOp and MOs cortical projections in other subcortical areas. (A–C) Representative images of spatial relationships along the dorsal-ventral axis in the STN (A), PG (B), and NOT (C). (D–F) Representative images of core-capsular pattern in the ZI (D), APN (E), and SCm (F). Bottom panels are zoomed, high-resolution images of the boxed areas. Scale bars: 1 mm (top) and 100 μm (bottom). Abbreviations can be found in Table 1.

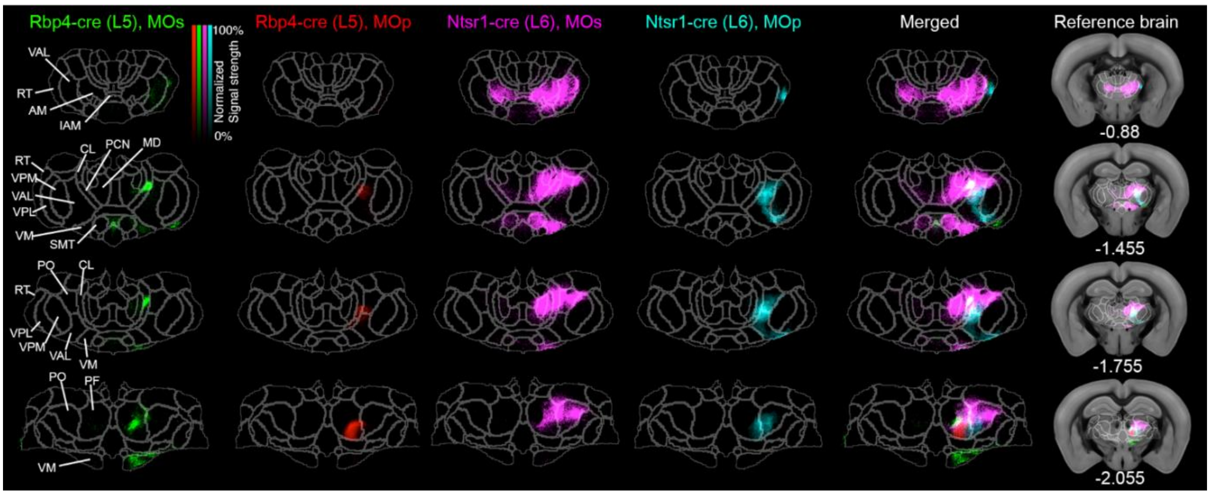

Supplementary Figure S3. Averaged projection signals in thalamic nuclei over reference STP brain anatomical labels. The numbers in the reference brain represent anterior/posterior bregma position; see also Supplementary Video 1 for the full dataset. Abbreviations can be found in Table 1. The heatmap represents normalized signal strength per voxel.

Virus labeled area	Anterior	Posterior	Medial	Lateral
Ntsr1, L6, MOp	1.34 ± 0.11	0.30 ± 0.24	1.76 ± 0.26	2.64 ± 0.26
Ntsr1, L6, MOs	1.88 ± 0.28	0.84 ± 0.22	0.74 ± 0.13	1.30 ± 0.12
Rbp4, L5, MOp	2.33 ± 0.18	0.38 ± 0.21	1.60 ± 0.14	2.88 ± 0.29
Rbp4, L5, MOs	2.33 ± 0.11	0.38 ± 0.19	0.62 ± 0.22	1.54 ± 0.09

Supplementary Table 1. Distances from the bregma of virus-expressing motor cortical areas in Rbp4-Cre and Ntsr1-Cre mice (n = 5 samples each) along the anterior-posterior and medial-lateral axes. Coordinate = mean ± standard deviation.

Supplementary Movie S1. Full projection map on the reference brain. Four different normalized and averaged projection signals overlaid on the RSTP brain. The top right movie contains bregma position information and the bottom right movie is the merged signal overlaid on an anatomical segmentation border. A detailed Allen Reference Atlas can be found at http://atlas.brain-map.org.

Supplementary Movie S2. Representative 3D tractome map of L5 motor cortical projections in Rbp4-Cre mouse. Each projection tract from MOp and MOs are maintained to their relative spatial positions but their innervation into subcortical areas are diversely distributed.

Supplementary Movie S3. Representative 3D tractome map of L6 motor cortical projections in Ntsr1-Cre mouse. L6 MOp projections are surrounded by L6 MOs projections in the thalamus. Cyan and Magenta are pseudo-color of tdTomato (Red) and GFP (Green) signals.
